# The influence of CYP 2C19*2 polymorphism on platelet function testing during single antiplatelet treatment with clopidogrel

**DOI:** 10.1186/1477-9560-9-4

**Published:** 2011-03-22

**Authors:** Alf-Aage R Pettersen, Harald Arnesen, Trine B Opstad, Ingebjorg Seljeflot

**Affiliations:** 1 Center for Clinical Heart Research. Department of Cardiology, Oslo University Hospital, Ullevaal, Oslo, Norway; 2 Faculty of Medicine, University of Oslo, Oslo, Norway

**Keywords:** Clopidogrel resistance, Functional tests, VerifyNow method, VASP method, Genetic polymorphisms

## Abstract

**Background:**

Different platelet function tests can be used to evaluate the degree of achieved platelet inhibition in patients treated with clopidogrel. The presence of CYP 2C19*2 polymorphism can reduce the formation of the active metabolite of clopidogrel, resulting in less platelet inhibition.

**Patients and Methods:**

Patients with symptomatic coronary artery disease, all on chronic single aspirin treatment were randomized to continue on aspirin or change to clopidogrel. In 219 randomly selected clopidogrel treated patients, platelet reactivity was evaluated by VASP-PRI determination and by use of VerifyNow P2Y12-PRU. The CYP 2C19*2 G/A polymorphism was further determined.

**Results:**

The total frequency of clopidogrel resistance was 29.0% by VASP-PRI and 31.6% by VerifyNow-PRU. The number of patients being hetero- and homozygous combined for the CYP 2C19*2 polymorphism (GA/AA) was 64 (29%). Platelet reactivity was significantly higher in patients with the polymorphism compared to wild-type patients (GG). VASP-PRI was 50.9% (SD19) in patients having the polymorphism compared to 38.3% (SD21) in patients with the GG genotype (p = 0.001). Correspondingly, the mean PRU was 165 (SD67) compared to 124 (SD69) (p < 0.001). The frequency of clopidogrel resistance in patients with the polymorphism was 32% compared to 16% in wild-type patients when defined by VASP-PRI (p = 0.006). When defined by PRU (VerifyNow), the corresponding frequencies were 53% and 22% (p < 0.001).

**Conclusions:**

Clopidogrel treated patients with the CYP 2C19*2 polymorphism have significantly increased platelet reactivity compared to patients with the wild-type, evaluated with the VASP determination, and even more pronounced with the VerifyNow P2Y12 method.

**Trial Registration:**

ClinicalTrials.gov: NCT00222261

## Background

Antiplatelet therapy is widely used in patients with a high risk of atherothrombosis and has become a cornerstone in treatment of coronary artery disease (CAD) [1]. Aspirin has been the primary choice for decades, whereas the benefit of adding clopidogrel in high-risk patients has been demonstrated in several trials [2,3]. Despite improvement in antiplatelet regimens, patients on-treatment run a considerable risk for new thrombotic events [4].

Although clinical benefit has been shown with clopidogrel, interindividual variation of platelet inhibition has been focused as an important explanation of insufficient platelet inhibition that might be a risk factor for new thrombotic events. The term "clopidogrel resistance" has been used to describe this phenomenon [5-7].

The in vivo transformation of clopidogrel to it's active metabolite is an important and critical step for the drug's antiplatelet effect. Clopidogrel is metabolized to the active metabolite that inhibits the ADP receptor P2Y12, and thereby inhibiting the ADP mediated platelet activation. This metabolization is dependent on the hepatic cytochrome P450 isoenzymes like CYP2C19, CYP1A2, CYP2B6, CYP2C9 and CYP3A4. Clopidogrel response variability is, to a large extent, explained by the extent of formation of the active metabolite of the drug [8].

Today, at least 25 single nucleotide polymorphisms (SNPs) in the gene coding for CYP2C19 have been described, and the "loss-of-function" allele CYP2C19*2 in exon 5, the most common and most studied polymorphism, has been shown to give a significant reduction in serum concentration of the active metabolite of clopidogrel and also to reduce inhibition of platelet aggregation [9-13].

Different platelet function tests have been used to evaluate the degree of achieved platelet inhibition in patients treated with clopidogrel. Light transmission aggregometry with ADP as an agonist, is the most evaluated and used method, but the test is time consuming and not practical for routine use. A new point-of-care system is the "VerifyNow" method in which the results have been shown to predict clinical outcome [14,15]. Determination of "Vasodilator Stimulated Phosphoprotein" (VASP) has been considered to be the most specific test for the degree of inhibition of the platelet P2Y12 receptor. Thus, this test has been considered to give the best answer on the platelet inhibition achieved by clopidogrel [16,17].

Several studies have reported on the platelet inhibiting effect of clopidogrel in combination with aspirin by use of different laboratory methods and cut-off values [7,18,19]. The aim of the present investigation was to study the frequency of resistance to single clopidogrel treatment in stable CAD patients by use of both the VerifyNow and the VASP methods. Further, we wanted to study the influence of the "loss-of-function" polymorphism, CYP2C19*2, on the functional assays.

## Materials and methods

### Study population

This is a sub-study of the Aspirin and Clopidogrel non-responsiveness clinical Endpoint Trial (ASCET) in which patients were enrolled between March 2003 and July 2008 [20]. The ASCET trial is a randomized, single center, open labeled clinical trial where 1001 patients with stable symptomatic CAD, all on chronic aspirin treatment, were randomized to either continued treatment with aspirin 160 mg/d or to clopidogrel 75 mg/d with a follow-up for two years. The study was approved by the regional ethics committee, and patients were included after giving their written informed consent.

In the present sub-study, all patients randomized to clopidogrel were consecutively included from October 2005 to June 2008 (n = 219).

In addition, patients from the ASCET cohort being on aspirin (n = 120), were included to identify the cut-off level for responsiveness with the VerifyNow- and VASP-methods. Recording of baseline characteristics were based on the patients medical files and the medical interview. Patients were classified as diabetics when previous diagnosed and treated diabetes or when presenting with fasting plasma glucose > 7.0 mmol/L. Hypertension was defined as previous diagnosed and treated hypertension. Recording of smoking habits were based on patient interviews. Previous smokers (smoking cessation more than 3 months ago) were classified as non-smokers. Current medication and body mass index (BMI) were recorded by patient interviews and by clinical examinations.

### Blood sampling

One month after randomization to clopidogrel, blood samples were drawn between 08.00 and 10.30 in fasting condition 24 hours after the last intake of medication. Compliance was assessed by interview and by a written questionnaire. Routine analyses were performed by use of conventional laboratory methods. Citrated blood (0.129 mM in dilution 1:10) was used for VASP analyses, and Vacuette tubes (Grüner Bio-One GmbH, Austria) (0.109 mM in dilution 1:10) were used for VerifyNow determination. For gene analyses, EDTA whole blood was used.

### VASP analysis

VASP is an intracellular actin regulatory protein. The phosphorylation of VASP is regulated by the cyclic adenosine monophosphate cascade. The phosphorylation status of VASP correlates with P2Y12 receptor inhibition. Thus, levels of VASP phosphorylation/de-phosphorylation reflect P2Y12 inhibition/activation.

VASP was determined, within 48 hours after blood collection, by use of the PLT VASP/P2Y12 assay (Biocytex, France). The FACS Calibur System (Becton Dickinson, Plymouth, UK) was used. The results are expressed as Platelet Reactivity Index (VASP-PRI) as described by the manufacturer. The lower the VASP-PRI, the higher is the biological effect of clopidogrel [17,21,22]. The intra assay coefficient of variation for VASP analyses was 2.3%.

### VerifyNow-P2Y12

VerifyNow (Accumetrics, San Diego, CA, USA) is an optically based detection device designed to measure platelet aggregation. This assay assesses the ability of activated platelets to bind fibrinogen-coated beads. In brief, the latter form mixed aggregates with stimulated platelets in whole blood in a process mediated by platelet GPIIb-IIIa receptors. ADP is incorporated to activate ADP receptors and prostaglandin E1 is added to reduce the non-specific contribution of the P2Y1 receptor. The instrument measures the change in light transmittance and the results are reported in Platelet Reaction Units (PRU). The intra assay coefficient of variation for VerifyNow analyses was 7%.

### Determination of Cut-Off values

We defined the cut-off levels for clopidogrel resistance as measured by VASP and VerifyNow P2Y12, as the lower 5 percentile of patients on chronic aspirin treatment (n = 120), giving VASP-PRI ≥55% and PRU ≥170 to be resistant.

### DNA isolation

DNA was purified from EDTA whole blood on the Magna Pure LC Instrument (Roche Diagnostics GmbH, Mannheim, Germany), using MagNA Pure DNA LC isolation kit, Large Volume (Roche Diagnostics GmbH). DNA purity and quantity were tested on the NanoDrop, ND-1000 (Saveen Werner, Sweden) and DNA was kept at -80°C until analysed.

### Genotype analysis

The "loss-of-function" cytochrome 2C19*2 G/A polymorphism *(rs 4244285) *was investigated. Allelic discrimination was performed by the ABI Prism 7900 HT Sequence Detection System using allele specific primers and probes included in the TaqMan Drug Metabolism Assay mix (Applied Biosystems, Foster City, CA, USA).

### Statistics

Continuous variables are presented as means ± SD and categorical variables are presented as numbers or percentages. Group comparisons were performed by Student's unpaired t-tests or Mann-Whitney U-tests when appropriate for continous variables and by the chi-square test or Fisher's exact test for categorical variables. Correlation analyses were performed by Spearmans rho. A p < 0.05 was considered to be statistically significant. SPSS statistical software, version 18.0 (SPSS Inc., IBM, Chicago, IL, USA) was used.

## Results

In Table 1, some selected clinical and laboratory characteristics of the total population (n = 219), all caucasians, are given. Number of samples successfully analyzed for the SNP was 218, 155 for VASP and 212 for VerifyNow, respectively.

**Table 1:** Baseline Characteristics of the Study Population

Age	(years)	62 ± 8.5
Male	(%)	79
Caucasian	(%)	100
SBP	(mmHg)	138.2 ± 18.6
DBP	(mmHg)	82.1 ± 9.2
Pulse	(beats/min)	61.7 ± 9.3
BMI	(kg/m^2^)	27.2 ± 3.7
Current smoking	(%)	16
History of hypertension	(%)	58
Diabetes mellitus	(%)	11
Previous PCI	(%)	38
Previous MI	(%)	37
Previous CABG	(%)	19
Total cholesterol	(mmol/L)	4.36 ± 0.94
LDL-cholesterol	(mmol/L)	2.38 ± 0.83
HDL-cholesterol	(mmol/L)	1.34 ± 0.36
Triglycerides	(mmol/L)	1.51 ± 1.12
***Medication:***		
Aspirin	(%)	100
Clopidogrel	(%)	0
Statins	(%)	98
Betablockers	(%)	71
CCBs	(%)	23
PPIs	(%)	14

Demographic and clinical characteristics of the study population (n = 219). Mean values ±SD and proportions are given.

Abbreviations: SBP (systolic blood pressure), DBP (diastolic blood pressure), BMI (body mass index), PCI (percutaneous coronary intervention), MI (myocardial infarction), CABG (coronary arterial by-pass grafting), CCBs (Calcium Channel Blockers), PPIs (proton-pump inhibitors)

### Functional tests

**Figure 1:**
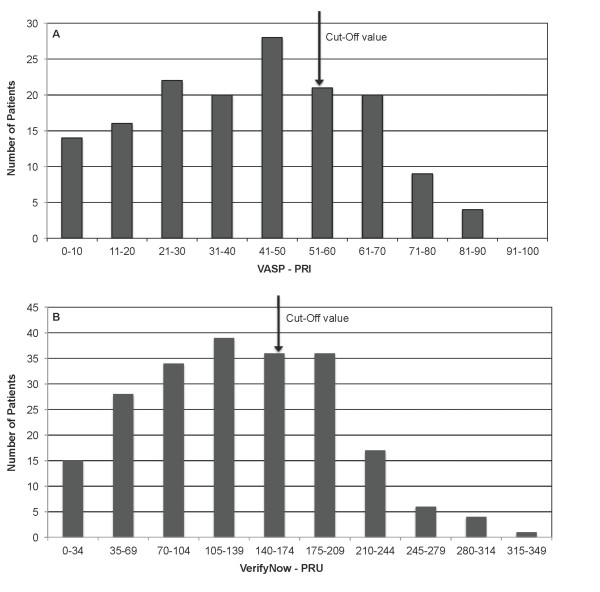
**Frequency distribution (in deciles) of VASP-PRI (Panel a) and VerifyNow-PRU (Panel b) in patients on clopidogrel as single antiplatelet therapy**. Cut-off levels ≥55 and ≥170, respectively, as indicated.

According to the defined cut-off values, the total frequency of clopidogrel resistance was 29% (n = 45) when measured by VASP determination and 32% (n = 67) when measured by the VerifyNow P2Y12. The distributions of response, shown in deciles in the total population, are shown in Figures 1a and 1b.

**Figure 2:**
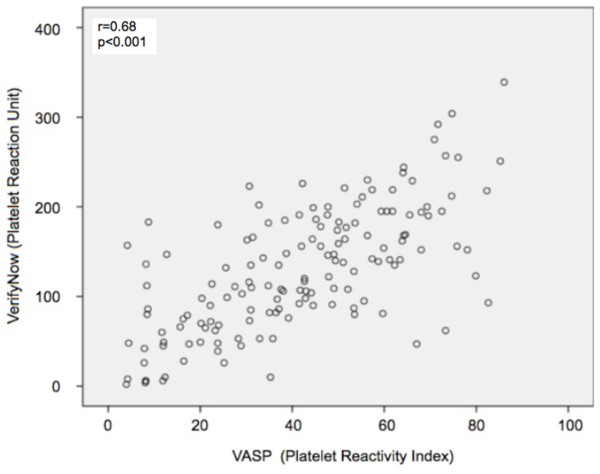
**The correlation between platelet reactivity index, PRI, measured by VASP and platelet reaction unit, PRU, measured by the VerifyNow**.

The correlation between the levels of VASP-PRI and VerifyNow-PRU was highly significant (r = 0.682, p < 0.001) (Figure 2).

Comparing the number of patients being resistant with the two methods, the concordance (agreement) was 74.5% (p < 0.001, kappa 0.379).

### Platelet response as related to genotype

The number of patients being heterozygous (n = 61) and homozygous (n = 3) combined for the CYP 2C19*2 polymorphism (GA/AA), was 64 (29%). There were no significant differences in any clinical characteristics between patients carrying the CYP2C19*2 polymorphism or not, except for a higher frequency of previous myocardial infarction in patients with the polymorphism (52% vs 32%, p = 0.004).

Platelet reactivity was significantly higher in patients with the polymorphism (GA/AA genotypes combined) compared to wild-type patients (GG) measured by both methods. Mean VASP-PRI levels were 51% versus 38%, and mean VerifyNow-PRU levels were 162 vs 121, respectively (p < 0.001 for both).

**Figure 3:**
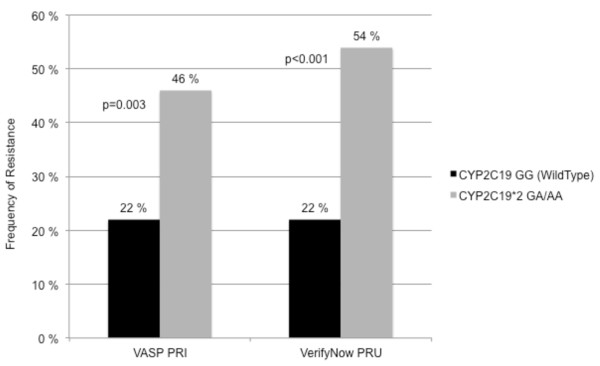
The frequencies of clopidogrel resistance in patients with and without the CYP2C19*2 A-allele, as determined by VASP-PRI and VerifyNow PRU.

Correspondingly, the frequency of clopidogrel resistance in patients with the polymorphism was 46% compared to 22% in wild-type patients when defined by VASP-PRI (p = 0.003) and 54% compared to 22% when defined by VerifyNow-PRU (p < 0.001) (Figure 3).

### Platelet response in relevant subgroups of patients (Table 2)

In the total population, there were no differences in the frequency of resistance within relevant subgroups, including use of medication, when assessed by VASP-PRI. Evaluated by VerifyNow PRU, higher prevalence of resistance was found in patients with previous myocardial infarction (p = 0.006) and in patients with BMI above median (27 kg/m^2^) (p = 0.015). Significantly higher frequency was also observed in calcium-channel blocker (CCB) users (45% versus 28%, p = 0.031). Specifically, no differences in the frequency of non-responders were observed between patients treated with proton pump inhibitors (PPI) or not (Table 2).

**Table 2:** Frequencies of clopidogrel resistance in subgroups

		VASP-PRI Resistant (n = 45)	**p-value **^**1**^	VerifyNow-PRU Resistant (n = 67)	**p-value **^**2**^
Diabetes	Yes	8 (31%)		14 (45%)	
	No	37 (29%)	.831	53 (29%)	.079
Smoking	Yes	7 (29%)		10 (30%)	
	No	38 (29%)	.987	57 (32%)	.861
Hypertension	Yes	27 (29%)		42 (35%)	
	No	18 (29%)	1.000	25 (27%)	.262
Previous MI	Yes	19 (33%)		34 (43%)	
	No	26 (27%)	.429	33 (25%)	.006
Statins	Yes	44 (29%)		63 (30%)	
	No	1 (25%)	.857	4 (80%)	.180
CCBs	Yes	10 (30%)		21 (45%)	
	No	35 (29%)	.856	46 (28%)	.031
PPIs	Yes	9 (43%)		7 (26%)	
	No	36 (27%)	.440	60 (32%)	.665
BMI≥27 (kg/m2)	Yes	29 (35%)		43 (39%)	
	No	16 (22%)	.082	24 (24%)	.015

Frequencies of clopidogrel resistance in subgroups, evaluated by VASP-PRI (n = 45 of 155) and VerifyNow-PRU (n = 67 of 212). Numbers (proportions) are given.

Abbreviations: MI (myocardial infarction), CCB (calcium channel blockers), PPI (proton pump inhibitors), BMI (body-mass index).

p^1^-values refer to differences in subgroups assessed by the VASP-method, and p^2^-values refer to differences in subgroups assessed by the VerifyNow-method.

## Discussion

In the present study, the main findings were that patients with stable CAD on single clopidogrel treatment carrying the CYP2C19*2 A-allele, had significantly higher prevalence of clopidogrel resistance measured by both the VASP and the VerifyNow methods. The concordance between the functional methods was only fair, although statistically significant.

We found no differences in the frequencies in demographic variables in patients having the "loss-of-function" polymorphism (GA/AA) or not, except for those presenting with a previous myocardial infarction, which was significantly more prevalent in patients with the A-allele. Whether this polymorphism per se increases the risk for atherothrombosis in clopidogrel-naive patients remains to be seen.

The presence of the "loss-of function" allele CYP2C19*2 is the most frequent and also the most studied polymorphism among the SNPs in the gene. It is reported that 25-30 percent of the US population is heterozygous while 3-4 percent is homozygous with no corresponding enzymatic activity, and similar prevalence of the SNP has been reported in a European population [12,13]. The frequency of this polymorphism in our study (29%) is in accordance with these reports.

Even though most of the other SNPs are rare, more studies are needed to elucidate the role of the less known polymorphisms of CYP2C19. It has recently been reported that carriers of CYP2C19*3 A-alleles, in addition to CYP2C19*2 A-alleles, have an increased risk for acute stent thrombosis [23]. One study indicates that there might be ethnical differences, and the "loss-of-function" CYP2C19*3 A-allele has been reported to be more frequent in an Asian than in US populations [24]. Clopidogrel treated patients carrying the CYP2C19*2 A-allele have been shown to have an increased risk for recurrent ischemic events [16,25,26]. On the contrary, the CYP2C19*17 has been associated with enhanced expression and enzymatic activity in the CYP2C19, representing a "gain-of-function" allele that might increase the risk of bleeding during clopidogrel treatment [12,27].

Several reports on platelet function testing in patients on clopidogrel treatment have shown large response variability and also an association to increased risk for recurrent ischemic events [4,10,11]. The clopidogrel response variability is mostly dependent on the extent of formation of the active metabolite in the hepatic CYP-system, although other mechanisms like intestinal absorbtion and platelet turnover may also play a role [12,28,29].

Determination of clopidogrel resistance as measured by both VASP-PRI and VerifyNow P2Y12 methods has been shown to predict clinical outcome [14-17]. However, there is still a need for standardization on how to test patients and how to define the cut-off levels. There are reports using cut-off values from 50 to 70% using the VASP-PRI method and from 162 to 235 units using the VerifyNow-PRU. We defined the cut-off values based on the lower 5 percentile in the ASCET study population, in which all are CAD patients, while on aspirin 160 mg/d. With these cut-off levels, we found that 29% were defined as resistant with the VASP method and 32% with the VerifyNow P2Y12 method. This correlates well with previous reports [22,30]. By using patients with documented CAD to estimate the cut-off value, we might achieve a lower cut-off value with a higher number of clopidogrel resistant patients because the control patients might have more activated platelets than healthy individuals. Controls from the same study population might therefore give a relevant picture.

The correlation between the two tests was highly significant, but the agreement between the tests was only fair (kappa 0.379). Using cut-off values of 70% (VASP-PRI) and 235 units (VerifyNow-PRU), the frequencies of resistance were reduced (Figure 1). The correlation and agreement between the methods were, however, improved (data not shown). Correlation with clinical end-points will obviously be of great importance for the evaluation of cut-off values.

Patients carrying the CYP2C19*2 A-allele had significantly higher prevalence of clopidogrel resistance measured by both VASP and VerifyNow. This is in line with previous reports [9,13]. However, most studies have been performed with use of only one of the methods and in patients on dual antiplatelet therapy with clopidogrel and aspirin. Carrying the polymorphism increases the risk for a reduced effect of clopidogrel, but it is important to keep in mind that 22% of the patients without the polymorphism (wild-types) were resistant when determining the platelet function (with both methods) and about 50% of patients with the polymorphism were responders (54% of patients with VASP and 46% with VerifyNow). Defining clopidogrel resistance with the genetic testing alone would give both a low sensitivity and specificity when identifying patients resistant to clopidogrel. Routine use of genetic testing is therefore so far not recommended [31,32]. Even though genotyping can be used to classify patients as poor or normal metabolizers, the actual platelet inhibition will be influenced by several other factors. Drug-drug interaction can alter the metabolization of clopidogrel to its active metabolite. Interaction with lipophilic statins, PPIs, calcium-channel blockers and warfarin, which all are metabolized by the CYP450, can reduce the formation of the active metabolite of clopidogrel, whereas cilostazol has been reported to increase the formation of the active metabolite [33-39]. Diverging results have been reported on the clinical relevance of the interaction between clopidogrel and PPIs. In our study, the use of PPIs did not influence the results of the platelet function tests, even in patients carrying the CYP2C19*2 A-allele. However, the number of patients using PPIs in our study was relatively low (n = 31 (14%)).

Patients on calcium-channel-blockers, achieved less platelet inhibition, with the VerifyNow method, than patients not treated with calcium channel blockers. These findings are in accordance with previous reports, and might be due to CCB inhibition of the cytochrome P450 3A4 enzyme, giving less formation of active metabolite from clopidogrel [38,40].

High BMI levels have also been shown to contribute to reduced clopidogrel response with increased platelet aggregation [41]. This is in line with our study, showing that patients with a BMI above median level had a lower degree of platelet inhibition compared to patients with BMI lower than median when measured by VerifyNow. It is not known whether there are specific mechanisms giving increased platelet aggregation in patients with a high BMI or if these findings are mainly a result of potentially confounding factors like diabetes, age and the lack of weight-adjusted maintenance doses of antiplatelet drugs [42,43]. Finally, smoking might induce the CYP450 activation, giving an increased platelet inhibition from clopidogrel [44,45]. However, in our study, there were no differences in platelet inhibition between smokers and non-smokers.

Different results were obtained with the VerifyNow and VASP methods in relation to differences in sub-groups of patients. The VerifyNow (P2Y12 cartridge) is measuring platelet aggregation with ADP as agonist, while VASP determination is a more direct measure of ADP inhibition caused by the active metabolite. This is also visualized in the relatively weak agreement between the methods. It might be suggested that VerifyNow in a better way express the platelets total aggregation potential.

This may possible be in line with our finding of an increased frequency of clopidogrel resistance in patients with previous MI when tested with the VerifyNow method.

## Conclusions

In the present study, patients with stable CAD on single antiplatelet treatment with clopidogrel had significantly reduced response when being carriers of the CYP 2C19*2 variant allele as compared to wild-type patients, when evaluated with the VASP method, and even more pronounced with the VerifyNow P2Y12 method. The consequences for clinical outcome are still debatable.

## Competing interests

The authors declare that they have no competing interests.

## Authors' contributions

AAP has been the main investigator of the ASCET trial and he has contributioned to design and performance of the trial, acquisition of the clinical data, analysis and interpretation of the data. AAP has drafted the manuscript.

HA has made substantial contributions to conception, design and performance of the trial, analysis and interpretation of the data. HA has helped to draft the manuscript.

TBO has made substantial contributions to the analysis and interpretation of the data. TBO has helped to draft the manuscript.

IS has made substantial contributions to conception, design and performance of the trial, analysis and interpretation of the data. IS has helped to draft the manuscript.
